# Interactive Effects of Perinatal BPA or DES and Adult Testosterone and Estradiol Exposure on Adult Urethral Obstruction and Bladder, Kidney, and Prostate Pathology in Male Mice

**DOI:** 10.3390/ijms21113902

**Published:** 2020-05-30

**Authors:** Julia A. Taylor, Maren Bell Jones, Cynthia L. Besch-Williford, Ashley F. Berendzen, William A. Ricke, Frederick S. vom Saal

**Affiliations:** 1 Division of Biological Sciences, University of Missouri, Columbia, MO 65211, USA; TaylorJA@missouri.edu (J.A.T.); marenbelljones@gmail.com (M.B.J.); 2 IDEXX BioAnalytics, Columbia, MO 65201, USA; Cindy-Besch-Williford@idexx.com; 3 Biomolecular Imaging Center, Harry S Truman VA Hospital and University of Missouri, Columbia, MO 65211, USA; ashley.berendzen@va.gov; 4 George M. O’Brien Center of Research Excellence, Department of Urology, School of Medicine and Public Health, University of Wisconsin, Madison, WI 53705, USA; rickew@urology.wisc.edu

**Keywords:** bisphenol A, diethylstilbestrol, estradiol, testosterone, obstructive voiding disorder, bladder obstruction, hydronephrosis, benign prostate hyperplasia, prostatitis

## Abstract

Obstructive voiding disorder (OVD) occurs during aging in men and is often, but not always, associated with increased prostate size, due to benign prostatic hyperplasia (BPH), prostatitis, or prostate cancer. Estrogens are known to impact the development of both OVD and prostate diseases, either during early urogenital tract development in fetal–neonatal life or later in adulthood. To examine the potential interaction between developmental and adult estrogen exposure on the adult urogenital tract, male CD-1 mice were perinatally exposed to bisphenol A (BPA), diethylstilbestrol (DES) as a positive control, or vehicle negative control, and in adulthood were treated for 4 months with Silastic capsules containing testosterone and estradiol (T+E2) or empty capsules. Animals exposed to BPA or DES during perinatal development were more likely than negative controls to have urine flow/kidney problems and enlarged bladders, as well as enlarged prostates. OVD in adult T+E2-treated perinatal BPA and DES animals was associated with dorsal prostate hyperplasia and prostatitis. The results demonstrate a relationship between elevated exogenous estrogen levels during urogenital system development and elevated estradiol in adulthood and OVD in male mice. These findings support the two-hit hypothesis for the development of OVD and prostate diseases.

## 1. Introduction

Obstructive voiding disorder (OVD) is common in aging men, and it is characterized by urgency, increased frequency of urination, hesitancy, low urine flow pressure, and incomplete bladder emptying, which can lead to acute kidney injury. OVD is associated with increased prostate size due to benign prostatic hyperplasia (BPH) or chronic prostatitis in approximately two-thirds of male cases, but OVD can also occur in the absence of any evidence of prostate enlargement [1]. Conversely, prostate disease does not always lead to OVD. Thus, the causes of OVD in men without prostate disease and in women, who can have similar symptoms at lower frequency [2], are at present unknown. However, we have hypothesized that the increase in serum estradiol that occurs as men age may be a significant factor in the etiology of OVD, since OVD is caused by administering estradiol to adult male mice [3].

We have found that exposure during the fetal period of urogenital sinus (UGS) differentiation to estradiol, as well as the estrogenic drugs diethylstilbestrol (DES) and ethinylestradiol (EE2), and to bisphenol A (BPA), an endocrine-disrupting chemical that has estrogenic activity and is used in many products, causes a decrease in diameter of the urethra and constriction of the bladder neck. All of these estrogenic compounds cause other gross UGS malformations, particularly in the colliculus region of the dorsal prostate in male mouse and rat fetuses [4,5,6].

Thus, there are two important windows of opportunity for estrogens to impact the development of OVD. These are the fetal–neonatal period, when the epigenetic programming of gene function occurs, leading to the perturbation of normal growth and development of the bladder, urethral, and prostate architecture, and then beginning in midlife, when glandular and stromal hyperplasia develops [3,7,8,9]. During aging, when testosterone production declines, men can also experience increasing levels of endogenous estradiol, which is positively related to body mass index, since fat cells contain the enzyme aromatase that converts androgens to estrogens [10]. In addition, it has been proposed that the decrease in the ratio of free testosterone to free estradiol, which is associated in men during aging with an increase in serum sex hormone binding globulin (SHBG) that binds testosterone with higher affinity than estradiol, is an important factor in the initiation of BPH and prostate cancer [11,12]. However, men are exposed not only to endogenous estrogens but also to environmental estrogenic chemicals, such as BPA, throughout life, and these contribute to the total estrogenic activity in estrogen receptor positive tissues.

We have published a series of studies showing that in adulthood, the treatment of male mice with testosterone and estradiol, but not testosterone alone, results in a significant constriction of the urethra as well as bladder outlet obstruction in treated males relative to controls; treated males showed enlarged bladders and droplet voiding. Estradiol and testosterone-treated males also showed prostate hyperplasia in the region of the prostatic ducts proximal to the urethra, which is the most sensitive region of the prostatic ducts to estrogen [3,13]. In addition, similar findings were reported for adult male mice treated with testosterone and BPA [14].

Prostate disease is influenced by estrogen exposure during urogenital tract development [9,15]. Periurethral glands develop throughout the urethra in male fetuses in humans, mice, and rats [16]. Prior studies have shown that prenatal exposure to very low doses of the estrogenic drugs DES and EE2, as well as BPA, stimulate an increase in the number and size of periurethral prostatic ducts in mice due to hyperplasia of the ductal epithelium, particularly in the dorsolateral region [5]. A similar increase in periurethral prostate gland size was seen in male rat and mouse fetuses exposed to elevated levels of endogenous estradiol due to being located in utero between female fetuses [17,18].

BPA exposure during fetal or adult life also resulted in the squamous metaplasia of the prostate, which involved the multilayering of basal epithelial cells and expression of cytokeratin 10 (CK10), which is a marker of squamatization in prostatic basal cells, in the anterior and dorsolateral prostate [8,19]. Developmental estrogen (estradiol, DES, EE2, and BPA) exposure was also associated with an increase in androgen receptor (AR) and estrogen receptor alpha (ERα) gene expression in UGS mesenchyme in fetal male mice [18,20,21,22,23], and increased prostatic androgen receptors and prostate size in the adult [4,20,24]. Thus, the increase in hormone receptor gene expression due to an increase in estrogen exposure during development is reflected by increased receptor content and a permanent increase in hormone sensitivity in adult life, with consequent changes to prostate function and disease.

Bisphenol A is a ubiquitous environmental estrogen to which virtually all people in the USA and elsewhere are exposed: over 90% of men and women in the USA are exposed to measurable levels of BPA [25], which can act additively with estradiol to induce estrogen-mediated responses [26,27]. Recent evidence suggests that prior assays used by the US Centers for Disease Control and Prevention (CDC) as well as the US Food and Drug Administration (FDA) to assess the concentration of total BPA in biomonitoring studies have dramatically underestimated actual concentrations, suggesting that the amount of exposure to BPA in the general population is much greater than previously assumed [28]. The high level of concern with BPA is based on the fact that it is one of the highest volume chemicals produced worldwide, generating about 20 billion US dollars in worldwide sales in 2020 and estimated to be over 30 billion dollars by 2026 [29].

This current work sought to investigate the potential interaction between developmental and adult estrogen exposure (a two-hit model) on the adult male urogenital tract, and we focus here on OVD and bladder and prostate size and pathology. We treated male CD-1 mice perinatally with low doses of the environmental estrogen BPA or the estrogenic drug DES as a positive control, and then administered testosterone and estradiol via Silastic capsules for 4 months in young adulthood. We show that adult slow-release hormone exposure elevated serum estradiol levels within a physiological range, which was comparable to that seen as a consequence of aging in men, while maintaining male reproductive organ function via combining the estrogen treatment with a physiological dose of testosterone shown to maintain accessory reproductive organ function in castrated males. The two-hit combined perinatal and adult exposures resulted overall in enlarged prostate as well as histopathology. We also observed bladder enlargement associated with urethral obstruction, leading in the most extreme cases to obstructive voiding disorder, inguinal hernias, massive accumulation of urine in the bladder, hydronephrosis, and death.

## 2. Results

### 2.1. Animal Group Designation, Sample Size, and Survival

We initially compared unhandled and oil-vehicle animals for all variables and found that the only difference was that the perinatal unhandled animals were 7.5% heavier than perinatal oil treated animals. Thus, the two perinatal control groups were combined into a single control group for comparison with the perinatal BPA and DES treatment groups. A randomly selected subset of these perinatal control animals and animals perinatally treated with BPA or DES received empty capsules in adulthood; these are hereafter referred to as CTL-0, BPA-0, and DES-0 groups, respectively. The other subset of perinatal control animals and animals perinatally treated with BPA or DES were implanted in adulthood with two Silastic capsules, one containing testosterone (T) and the other containing estradiol (E2); these groups are referred to as CTL-TE2, BPA-TE2, and DES-TE2 groups, respectively. The number of males in these 6 groups was: CTL-0 n = 19 from 14 litters, BPA-0 n = 16 from 11 litters, DES-0 n = 19 from 11 litters, CTL-TE2 n = 22 from 14 litters, BPA-TE2 n = 13 from 11 litters, and DES-TE2 n = 18 from 11 litters. A few of these males did not survive to the end of the experiment. For males treated in adulthood with empty capsules, only 1 treated male (perinatally with BPA) died of an unknown cause. The number of males treated in adulthood with T+E2 that died before the end of the 4-month adult treatment period was: CTL-TE2 = 2 males, BPA-TE2 = 2 males, DES-TE2 = 4 males. In most cases, the deaths for T+E2-treated males was associated with enlarged bladders, and they included inguinal hernias and kidney abnormalities. All other males were included in the analyses.

### 2.2. Serum Estradiol Concentrations

**Figure 1:**
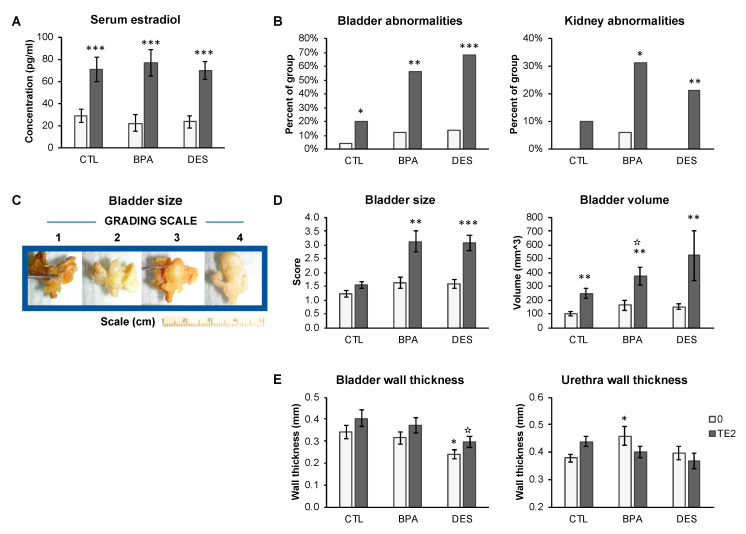
Serum estradiol concentrations and bladder, kidney, and urethra metrics in control (CTL-), BPA-, or DES-treated mice, without (0, light bars) and with (TE2, dark bars) adult treatment. (**A**) Serum estradiol concentrations and (**B**) incidence of bladder and kidney abnormalities observed at the time of necropsy. (**C**) Examples of bladder size using the observational grading scale. (**D**) Bladder size based on observational grading (left) or on volume calculated from external dimensions (right) in fixed tissues. (**E**) CT measurements of bladder wall thickness and post-prostatic urethra wall thickness in fixed tissues. Values are mean ± SEM. * *P* < 0.05, ** *P* < 0.01, *** *P* < 0.001 vs. CTL-0; ✫ *P* < 0.05 vs. CTL-TE2.

Serum estradiol was measured by radioimmunoassay in a subset of each treatment group (*n* = 9–13 per treatment). Serum estradiol concentrations for animals with empty capsules (overall mean for all treatment groups ± SEM) was 25.5 ± 5.2 pg/mL, which was significantly lower (*P* < 0.001) than for animals from different perinatal treatment groups with T+E2 capsules, where the overall mean (±SEM)) was: 68.3 ± 5.3 pg/mL. The planned comparison of males with empty capsules versus T+E2 capsules within each perinatal treatment group (CTL-0 versus CTL-TE2; BPA-0 versus BPA-TE2, and DES-0 versus DES-TE2) were all statistically significant (*P* < 0.005). Thus, the estradiol assay confirmed elevated exposure to estradiol within a physiological range due to T+E2 treatment (Figure 1A).

### 2.3. Bioassays for Release from Silastic Capsules of Testosterone and Estradiol

Testosterone and estradiol release from Silastic capsules (at the doses used in the experiment) was confirmed by bioassay in small groups of untreated adult male and female mice. Uterine weight in gonadectomized female mice averaged 54.4 ± 14.7 mg, whereas gonadectomized females that received estradiol via Silastic capsules (at the dose used in the present experiment) had a mean (±SEM) uterine weight of 251.2 ± 13.6 mg (*P* < 0.001), which was similar to our previously reported uterine weight in response to treatment with this dose of estradiol [30]. At 2 weeks post-castration, seminal vesicle weights of male mice were less than half those of gonadally intact mice (28.4 ± 3.3 mg versus 63.4 ± 5.7 mg, respectively; *P* < 0.01). For gonadectomized males receiving testosterone via Silastic capsules, the average seminal vesicle weight was 76.6 ± 5.2 mg, which did not differ significantly from seminal vesicles of gonadally intact males.

### 2.4. Bladder and Urethra Necropsy Findings

At the time of sacrifice, we repeatedly observed the same pathologies, predominantly, although not exclusively, in animals treated with BPA or DES during perinatal life and then exposed to T+E2 capsules in adulthood. Notably, we observed enlarged bladders that did not appear to be able to void at the time of necropsy, enlarged and/or discolored kidneys and hydronephrosis (Supplemental Figure S1). Abnormal bladders, referring to any abnormality of the bladder at necropsy (essentially an enlarged bladder with or without herniation (Figure 1B), were present in 20%–68% (depending on perinatal treatment) of animals with adult T+E2 capsules. Herniated bladders protruding into the inguinal canal were always enlarged. Incompletely voided bladders were seen in only one CTL-0 animal, 2 BPA-0 animals, and 3 DES-0 animals. T+E2 treatment in adulthood increased the incidence of bladder abnormalities (*P* < 0.05, *P* < 0.01 and *P* < 0.001 for Oil-TE2, BPA-TE2, and DES-TE2 males, respectively. With one exception (kidney discoloration was found in one BPA-0 animal), kidney abnormalities (discoloration, enlargement and hydronephrosis) were not seen in animals treated with empty capsules, and they were significantly increased by adult T+E2 treatment in perinatally exposed BPA and DES males (*P* < 0.05, *P* < 0.05, *P* < 0.01 for Oil-TE2, BPA-TE2 and DES-TE2 males, respectively Figure 1B).

We measured bladder volume (Figure 1C) from CT scans using the Siemens Inveon software, which permits 3D volume calculations. This proved to be problematic owing to the fact that the large empty bladders tended to flatten and wrinkle. Thus, we also used another approach, which was to measure the external dimensions manually and compute bladder volume mathematically (see Methods), and these values showed a reasonable correlation with those from the CT scan (R^2^ = 0.607; Supplemental Figure S2). Bladder size and volume based on our blinded observational (manual) grading are shown in Figure 1C. Bladder size measured by observational grading was significantly increased by adult T+E2 capsule treatment in both BPA- and DES-treated animals (for both approaches to measurement, CTL-0 versus BPA-TE2, *P* < 0.01; CTL-0 versus DES-TE2, P<0.001), but not in CTL-0 versus CTL-TE2-treated animals (*P* = 0.1). Perinatal treatment did not significantly affect bladder size in animals that were not exposed to adult T+E2. Bladder volume calculated from external bladder dimensions (also measured blinded) was significantly increased by T+E2 treatment in adulthood for all perinatal treatment groups (*P* < 0.01), but again, it did not differ significantly for animals with empty capsules in adulthood, regardless of perinatal treatment (Figure 1D). Sample sizes for this analysis were CTL-0 n = 7, BPA-0 n = 15, DES-0 n = 19, CTL-TE2 n = 6, BPA-TE2 n = 7 and DES-TE2 n = 8.

**Figure 2:**
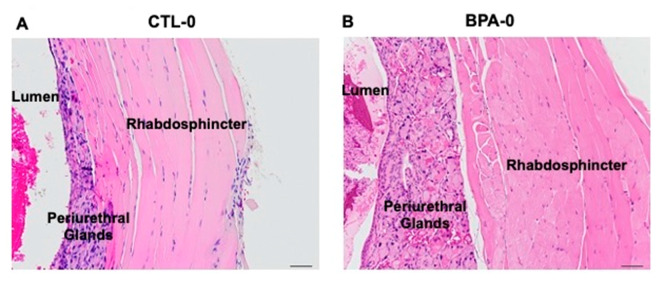
Post-prostatic urethra between the prostatic and penile urethra. Hematoxylin and eosin stained sections of the urethra wall from adult male mice. (**A**) CTL-0 male, showing normal lumen and urethra wall: periurethral mucus glands, rhabdosphincter muscle. (**B**) BPA-0 male showing thickened urethra wall due to glandular hyperplasia and an increase in thickness of the rhabdosphincter (20× magnification).

Bladder wall thickness measured from CT scans (Figure 1E) was significantly increased by adult T+E2 treatment relative to males treated with empty capsules when ignoring perinatal treatment group (0.30 ± 0.02 mm versus 0.36 ± 0.02 mm, *P* < 0.05, respectively). In contrast, bladder wall thickness was reduced in DES-TE2 males compared to CTL-TE2 males and also CTL-0 versus DES-0 males (*P* < 0.05 for both comparisons; Figure 1E). The thickness of the post-prostatic urethra wall was significantly increased by perinatal BPA alone (CTL-0 versus BPA-0, *P* < 0.05; Figure 1E). T+E2 treatment tended to increase urethra wall thickness in CTL-TE2 males compared to CTL-0 males, but this was not statistically significant (*P* = 0.07), and T+E2 treatment did not significantly affect urethra wall thickness in BPA or DES animals. The overall group data for urethra wall thickness (Figure 1E) was complicated by bladder status. In histological sections of selected representative animals (not shown), the urethral wall was thickened in a CTL-TE2 animal with an obstructed bladder but not in a CTL-TE2 animal with a normal bladder. Obstructed BPA-0 and BPA-TE2 animals had urethral wall thickening (Figure 2), but a BPA-TE2 animal with a normal bladder did not. Generally, stromal thickening or hyperplasia was also observed in sections from bladder-obstructed animals regardless of perinatal treatment (Figure 2), but not in sections from animals with normal bladders. We had intended to also examine the prostatic urethra, but the CT scans did not provide the resolution required for accurate measurement of the prostatic urethra due to the complexity of the architecture of this region of the urethra.

### 2.5. Perinatal BPA Plus Adult T+E2 Impacts the Dorsal Prostate as Well as Urine Retention in the Bladder

**Figure 3:**
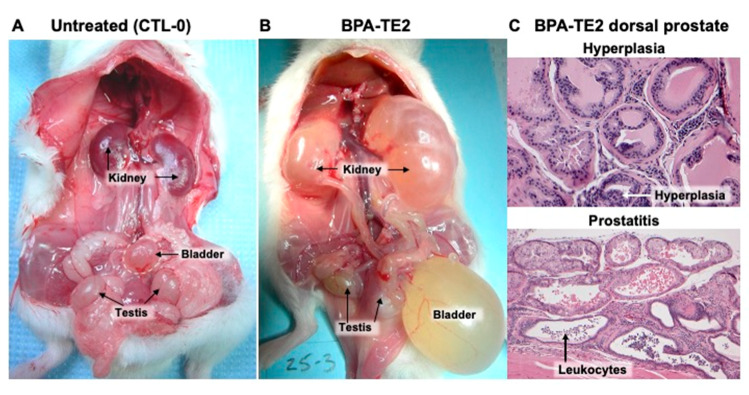
(**A**) Normal urogenital system in a perinatal control male mouse treated in adulthood with an empty capsule (CTL-0). (**B**) Bladder obstruction and hydronephrosis in an animal exposed perinatally to bisphenol A (BPA) and given testosterone and estradiol (T+E2) treatment in adulthood, and (**C**) dorsal prostate (with hematoxylin and eosin staining) showing epithelial hyperplasia and leukocyte infiltration of prostatic ducts (indicative of prostatitis) in the same BPA-TE2 animal shown in B (40× magnification).

**Figure 4:**
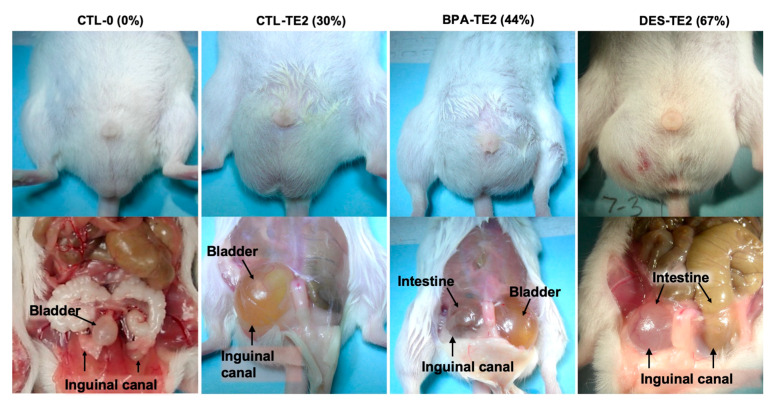
Examples of the effect of T+E2 treatment on inguinal hernias observed in this study, also showing the percent of males within each T+E2 group with inguinal hernias (only T+E2-treated males showed inguinal hernias). CTL-0 is an untreated normal male, and the lower panel shows the absence of an inguinal hernia. CTL-TE2 is a perinatal untreated male exposed to T and E2 in adulthood. The top panel shows the wetness of fur around the penis indicative of droplet voiding, and the bottom panel shows an enlarged bladder in the right inguinal canal. BPA-TE2 is a male treated perinatally with BPA and then T and E2 in adulthood. The top panel shows the wetness of fur around penis, and the bottom panel shows an enlarged bladder herniated on the left side and the cecum herniated on the right side. DES-TE2 is a male treated perinatally with diethylstilbestrol (DES) and then T and E2 in adulthood. The top panel shows bulging in the scrotal region, and the bottom panel an intestinal herniation in the right and left inguinal canals. The incidence of hernias was significantly increased by T+E2 treatment for all perinatal treatment groups (*P* < 0.01).

An extreme example of a BPA-TE2 male (Figure 3B) with obstructive voiding disorder and a severely distended bladder full of urine and hydroureteronephrosis is shown in comparison to a CTL-0 normal male in (Figure 3A); this and other obstructed males had wet fur on the belly/prepuce area (Figure 4), suggesting urine dribbling indicative of obstructive voiding disorder [3]. The dorsal prostate glands in this obstructed BPA-TE2 male showed ductal epithelial hyperplasia and leukocytic invasion (indicative of prostatitis) on histological examination (Figure 3C).

### 2.6. Inguinal Hernias

Inguinal hernias were seen exclusively in T+E2-treated animals, affecting 30% of CTL-TE2, 44% of BPA-TE2, and 67% of DES-TE2 animals (Figure 4). The incidence of hernias was significantly increased relative to CTL-0 by T+E2 treatment (*P* < 0.01, *P* < 0.01, *P* < 0.05 for CTL-TE2, BPA-TE2 and DES-TE2, respectively). In some of the animals affected by herniation (n = 3–4 per treatment group), measurements of inguinal canal diameter were made after withdrawal of the intruding tissue. With the exception of one animal, in which the right inguinal canal diameter was 10.2 mm, inguinal canal measures were very similar at around 6 mm diameter in all treatment groups, with or without adult hormone treatment, suggesting that neither perinatal treatment nor adult hormone exposure specifically affected inguinal canal diameter. Males with inguinal canal hernias containing bladder and/or intestine typically had minimal adipose tissue in the gonadal fat pads (Supplemental Figure S1).

### 2.7. Prostate Size

**Figure 5:**
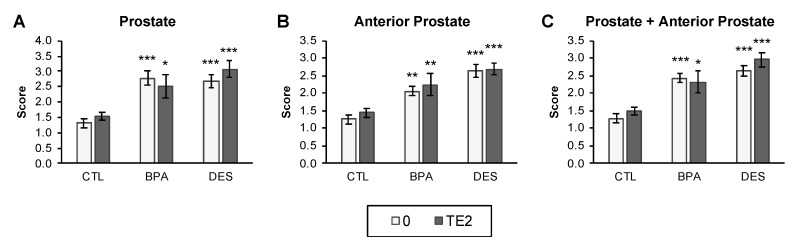
Assessment of prostate size (conducted blind) by observational grading. (**A**) Prostate size (dorsal, lateral, and ventral lobes), (**B**) anterior prostate size, and (**C**) size of the combined prostate and anterior prostate. Values are mean ± SEM of scores using a 4-point scale similar to that used for the bladder (see Figure 1 and Methods). * *P* < 0.05, ** *P* < 0.01, *** *P* < 0.001 vs. control males (CTL-0).

Prostate (dorsal, lateral, and ventral lobes), anterior prostate (coagulating gland), and seminal vesicles were scored on a 4-point scale by an investigator blind to the treatment group. Prostate size was significantly increased by perinatal BPA and DES treatment (*P* < 0.05 for both CTL-0 versus BPA-0 and CTL-0 versus DES-0). The T+E2 capsule had no further effect on relative prostate size, and compared to CTL-0, both BPA-TE2 and DES-TE2 males had significantly larger prostates (Figure 5). The anterior prostate gland size followed a similar pattern to that of the rest of the prostate, as did the combined prostate and anterior prostate, with perinatal influence of BPA and DES on gland size (CTL-0 versus BPA-0, *P* < 0.05; CTL-0 versus DES-0, *P* < 0.01). No additional effects of T+E2 capsule treatment were seen for the anterior prostate, as was the case for the dorsal, lateral, and ventral prostate lobes.

Prostate sections stained with hematoxylin and eosin (H&E) from CTL-0 and CTL-TE2 animals were largely unremarkable, regardless of bladder status, as were sections from BPA-0 and DES-0 animals. For the T+E2-treated BPA and DES animals, there were differences between sections from animals with normal versus obstructed bladders. Prostatic glands were malformed in the bladder-obstructed DES-TE2 animal, but other groups appeared normal on gross examination. In BPA-TE2 and DES-TE2 animals with normal appearing bladders, prostatic glands appeared histologically normal with H&E staining, but in the BPA-TE2 and DES-TE2 animals with bladder obstruction, there was evidence of prostatic epithelial hyperplasia as well as prostatitis (Figure 3C). Seminal vesicle size was significantly reduced by perinatal DES treatment compared to CTL-0 males, for both DES-0 and DES-TE2 males (*P* < 0.05), similar to prior findings that developmental exposure to elevated estradiol decreased seminal vesicle size due to a permanent reduction in 5α-reductase activity [31]. However, seminal vesicle size was not significantly different for BPA-treated males (data not shown).

### 2.8. Other Organ Weights at Necropsy

Necropsy body weight and organ weight data for the three treatment groups with and without adult T+E2 treatment are shown in Supplemental Figure S1. There were no treatment effects on body weight. However, this result is difficult to interpret, because some of these animals were clearly not healthy, and the retention of urine in T+E2 males likely made them appear heavier, since treatment with estrogen in adulthood reduces body weight. For example, significantly reduced gonadal fat pad weight was found in BPA-TE2 and DES-TE2 males (BPA-0 versus BPA-TE2, *P* < 0.05; DES-0 versus DES-TE2, *P* < 0.05). However, compared to the CTL-0 males, DES-0 males also had significantly reduced gonadal fat pad weight (*P* < 0.05).

Disregarding perinatal treatment, preputial gland weight was significantly (*P* < 0.001) reduced by adult T+E2 treatment by 24% compared to CTL animals (0.1219 ± 0.0080 g versus 0.1602 ± 0.0074 g, respectively). Compared to CTL-0 males, preputial gland weight was significantly reduced in CTL-TE2, BPA-TE2, and DES-TE2 males Supplemental Figure S1). Adult treatment with T+E2 as well as perinatal BPA or DES treatment also resulted in some preputial glands being abscessed or atrophied, and a few were inflamed (Supplemental Figure S3).

Kidney weight was significantly increased in BPA-TE2 and DES-TE2 males compared to the same perinatal treatment but without adult TE2 treatment (*P* < 0.05), while TE2 treatment did not have an effect on kidney weight in CTL males (Supplemental Figure S1). This reflected the kidney disease status of the animals perinatally treated with BPA or DES and then treated with T+E2 in adulthood (Figure 1B and Figure 3B).

### 2.9. Daily Sperm Production and Testis Weight

Daily sperm production (DSP) was measured in the right testis from randomly selected subsets of perinatally and adult-treated animals (*n* = 7–8 per treatment). Disregarding perinatal treatment, daily sperm production was significantly reduced by T+E2 treatment in adulthood relative to males with empty capsules (4.11 ± 0.34 versus 5.92 ± 0.32 (×10^6^) sperm/day, *P* < 0.001, respectively). Relative to perinatal control males implanted with empty capsules in adulthood (CTL-0 males), CTL-TE2, BPA-TE2 or DES-TE2 treated males had significantly reduced DSP (*P* < 0.01; Supplemental Figure S4A). No significant differences in DSP were observed between CTL-0 and BPA-0 or DES-0 animals. The reduced DSP associated with adult T+E2 treatment was associated with reduced testis weight, which was significantly reduced by T+E2 treatment for all perinatal groups relative to CTL-0 males (*P* < 0.001; Supplemental Figure S4B).

### 2.10. Differences in Serum Estradiol in T+E2-Treated Males Impacted Phenotype

**Figure 6:**
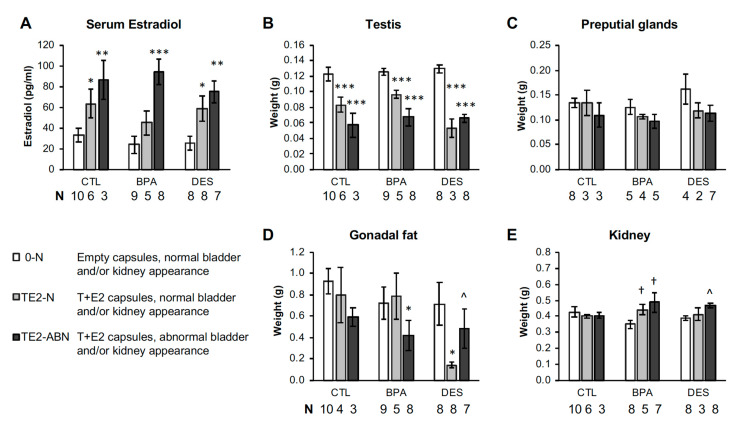
Serum estradiol and organ/tissue weights at necropsy in males without or with obstructed bladders and/or kidney abnormalities based on observational criteria (conducted blind to treatment group). (**A**) Serum estradiol concentrations in CTL, BPA, and DES animals with normal (N) or abnormal (ABN) bladder and/or kidneys. (**B–E**) Organ weights in the same animals (right testis, combined preputial glands, combined gonadal fat pads and right kidney). Values are mean ± SEM. ^ *P* = 0.07, * *P* < 0.05, ** *P* < 0.01, *** *P* < 0.001 vs. CTL-0-N; † *P* < 0.05 vs. the same perinatal treatment without T+E2 in adulthood.

Since we were interested in the relationship between adult serum estradiol and the incidence of bladder, kidney, prostate, and other pathologies, we examined serum E2 and the weights of tissues in animals exhibiting bladder and kidney pathologies, and these data are presented in Figure 6. For convenience, and because physiologically the two pathologies are often related, we examined the animals with bladder obstruction and/or kidney abnormalities as a single group. The CTL, BPA, and DES groups shown in Figure 6 are separated into 0-N (empty capsule, normal bladder, and kidneys), TE2-N (T+E2 capsules, normal bladder and kidneys) and TE2-ABN (T+E2 capsules, abnormal bladder and/or kidneys). Since serum E2 was only measured in the subsets of each treatment group, Figure 6 only includes those animals for which there was a serum E2 measurement, and the sample sizes for the organ weights are consequently smaller.

T+E2-treated animals with bladder and/or kidney abnormalities had the highest serum E2 concentrations, while T+E2 males in all perinatal treatment groups with normal bladders and kidneys had lower serum E2 levels. Serum E2 concentrations were related to right testis, gonadal fat pad, and right kidney weight, and they had a modest, but not statistically significant, effect on preputial gland weight (Figure 6).

## 3. Discussion

The results of this study with male CD-1 mice demonstrate an interaction between perinatal exposure to BPA (20 µg/kg/day, p.o.) or DES (0.2 µg/kg/day, p.o.) and adult exposure to combined testosterone (T) and estradiol (E2), at physiologically relevant levels, on bladder, kidney, and prostate abnormalities (Figure 1B,D). Prostate abnormalities related to this “two-hit” estrogen exposure model included epithelial hyperplasia and prostatitis in the dorsal prostate (Figure 3C), which was associated with urine retention in the bladder, indicating obstructive voiding disorder (OVD), and kidney damage (hydronephrosis; Figure 3B). There was also an increase in the size of the combined dorsal, lateral, and ventral prostate as well as the anterior prostate caused by perinatal exposure to BPA or DES that occurred with or without adult T+E2 treatment (Figure 5). Perinatal exposure to BPA, which is an environmental estrogen (but also has other hormonal/antihormonal activities), or the drug DES, thus appeared to affect the sensitivity of the male urogenital system to adult estrogen exposure, and the similarity of BPA and DES findings suggest an estrogenic mode of action.

### 3.1. Obstructive Voiding Disorder

In more detail, adult exposure to T+E2 via Silastic capsules for 4 months was associated with evidence of OVD, regardless of prior perinatal treatment, but the effects were greatest in males treated perinatally with BPA or DES (Figure 1). The adult T+E2 treatment findings confirm our prior findings of enlarged bladders and OVD following adult treatment alone in C57BL/6J male mice with T and E2 pellets reported by Nicholson et al. [3]. However, the CD-1 male mouse appears less sensitive to adult T+E2 than the C57 unless there is prior perinatal BPA and DES sensitization of the male urogenital system to adult androgen and estrogen exposure; these are referred to as “organizational” and “activational” effects of gonadal steroids, respectively [32].

We previously reported [3] thinned bladder walls and narrowing of the prostatic urethral lumen following just adult exposure to T and E2. In the present study, T+E2 treatment increased bladder wall thickness relative to males from the same perinatal treatment group, unlike the findings with C57 mice by Nicholson et al. However, we did observe bladder wall thinning in perinatal DES animals without adult T+E2 treatment relative to CTL-0 males (Figure 1E). Our measurements of the post-prostatic urethra (in a region distal to the prostate) did indicate thickening of the urethral wall in BPA-0 animals treated in adulthood with empty capsules relative to CTL-0 males, but not in DES-treated males. Histologically, we observed thickened post-prostatic urethra walls (periurethral glandular hyperplasia and enlarged rhabdosphincter) in BPA-0 animals not exposed to T+E2 in adulthood, suggesting a predisposition to bladder obstruction (Figure 2). Our prior work [5] had shown constriction of the urethra at the bladder neck in CD-1 mouse fetuses exposed to similar low doses of BPA or DES used in the present study.

### 3.2. Modulation of Androgen Receptors (AR) and Estrogen Receptors (ER)

We and others have shown that AR and ERα are upregulated in CD-1 male fetal UGS mesenchyme by estrogenic chemicals, including BPA and DES [9,20,21], and that in adulthood, this leads to upregulated AR protein [4,20]. Other findings suggest that perinatal exposure to low, physiologically relevant doses of estrogenic chemicals, such as DES, leads to the upregulation of ERα protein expression in the uterus in adulthood, which is associated with an increase in uterine weight and an increase in ERα-mediated responses; in contrast, high doses of DES used in toxicological experiments have the opposite effect and result in the disruption of uterine morphology and function [33]. This is the same nonmonotonic outcome that we have reported for consequences of developmental exposure to low vs. high doses of DES in the fetal urogenital system and adult prostate in male mice [4,5]. Generally, the lifetime expression of steroid receptors is impacted by the epigenetic control systems that are “organized” by endogenous and manmade hormones during the perinatal period of sexual differentiation in rodents and other animals, with hormone dose being a critical factor [22,34]. The epigenetic mechanisms that mediate these effects are complex and as yet are not fully understood [23].

### 3.3. Inguinal Hernias

A change in incidence of hernias as well as bladder and kidney abnormalities was not induced by perinatal treatment alone in that the hernias did not occur in CTL-0, BPA-0, and DES-0 animals. The incidence of hernias was significantly increased by treatment in adulthood with T+E2 regardless of perinatal treatment, but it was higher in BPA-TE2 and DES-TE2 males in comparison to CTL-TE2 males (Figure 4). Zhao et al. [35] reported that an increase in estradiol resulted in fibrosis in the lower abdominal muscle, which led to inguinal hernias in a mouse model. Our findings are consistent with an increase in estradiol being involved in the etiology of inguinal hernias, which is one of the most common surgeries performed in the USA [36]. We did notice that animals with hernias tended to have smaller gonadal fat pads, and we propose that a contributor to herniation might be a reduced barrier to the inguinal canal, along with effects on abdominal muscle reported by Zhao et al.

### 3.4. Prostate Pathology

Perinatal BPA or DES exposure resulted in increased adult combined dorsal, lateral, and ventral prostate (as well as anterior prostate) size, with or without adult treatment with T+E2 (Figure 5), similar to our prior reported effects of low dose prenatal estradiol, BPA, DES, and ethinylestradiol exposure on adult prostate weight in male mice [4,24,37]. Histologically, the prostates examined in a subset of BPA-TE2 and DES-TE2 males with normal bladders did not appear grossly abnormal, but those with bladder obstruction showed epithelial hyperplasia and prostatitis in the dorsal prostate. In our prior studies, the dorsal prostate has been the most affected by developmental exposure to estrogenic chemicals [5].

In the Noble rat that is a well-studied model for the hormonal induction of prostate cancer, extended T+E2 treatment in adulthood resulted in prostatic hyperplasia by 2 months, the first appearance of prostate carcinoma within 4 months, and prostate cancer in over 90% of males after 12 months of continuous exposure of T+E2 via Silastic capsules (the same approach used by us but at higher doses [38]). Nicholson et al. [3] treated C57 male mice with T+E2 pellets and saw enlarged prostates at 4 months as well as clear evidence of OVD, but the mice did not show evidence of prostatic cancer induction. Thus, our results are not consistent with the C57 mouse study in that we did not see prostate enlargement in T+E2-treated CTL animals in the CD-1 male mouse. Differences between the study results may be due to strain differences in sensitivity to these sex hormones, lower levels of serum T+E2 concentrations in our present study, or to differences in how prostate size was measured.

### 3.5. Estrogen, Obstructive Voiding Disorder, and Prostate Pathology

Discussion of OVD often centers on associated benign prostatic hyperplasia (BPH) as a causative or at least contributory factor, but about one-third of men over age 65 with lower urinary tract symptoms have few or no symptoms of BPH [39]. Although we determined histologically that bladder obstruction was associated with prostate disease, our histological examination focused on a small sample of animals, and thus we could not determine whether all animals with pronounced bladder obstruction also had prostate pathology. However, for both BPA and DES animals, prostate size was increased relative to controls, with no apparent additional effect of adult hormone treatment, while bladder volume was only increased in T+E2-treated adult animals. Thus, at least for empty capsule treatments in adulthood, in BPA and DES-exposed animals, prostate size was increased with no effect on bladder volume, which required adult T+E2 treatment (a second hit). Developmental exposure to other endocrine disruptors, such as dioxin, in conjunction with adult T+E2 exposure, also leads to prostate disease and lower urinary tract dysfunction in male mice [40]. These findings suggest that other endocrine disrupting chemicals may also be involved in the etiology of these diseases.

Serum E2 concentrations were elevated in animals receiving the T+E2 capsules and averaged 68.3 ± 5.2 ng/mL, which is a value similar to that in late proestrous female mice, but individual values varied, which Figure 6 showed was related to variability in pathology incidence in that not all T+E2 animals developed enlarged bladders or one of the other co-pathologies. The T+E2-treated animals with urinary tract anomalies had the highest E2 values (Figure 6).

In prior work [41], we reported increased gonadal fat pad cell number and volume, increased serum insulin, and serum glucose during both insulin-tolerance and glucose-tolerance tests in CD-1 male mice exposed prenatally to a maternal dose of BPA of 5 µg/kg/day, which resulted in average fetal serum levels of free BPA of 2 pg/mL between GD 17–19. This provided evidence for significant effects of very low doses of BPA that are assumed to be safe by the FDA on metabolic control systems. In the present perinatal exposure study, BPA exposure was continued throughout the early postnatal period (up to postnatal day (PND) 15) of prostate differentiation [42], and we saw a small but not statistically significant increase in body weight in BPA (but not DES) treated males not exposed to T+E2 in adulthood (Supplemental Figure S1). T+E2 treatment significantly reduced gonadal fat pad weight in both BPA and DES animals in this study to a greater degree than perinatal controls exposed to T+E2, suggesting a greater sensitivity to adult T+E2 treatment in the BPA and DES-treated animals. T+E2 treatment also decreased daily sperm production and testis weight (Supplemental Figure S4). An interesting feature of estrogens is that their developmental effects are to a large degree opposite to their effects in adulthood on metabolic systems, such as the control of adipocyte function [43].

Of great interest are the effects of developmental exposure to BPA and other xenoestrogenic chemicals on epigenetic mechanisms and signaling systems involved in differentiation of the urogenital system in males [22,23], which will require much more research to uncover. The molecular mechanisms in the male urogenital system that are impacted by fetal–neonatal estrogenic chemical exposures, and their interaction with subsequent adult estrogen exposure in the etiology of disease during aging is an active area of investigation in rodent models [15]. The present study identified a phenotype caused by combined xenoestrogen treatment during development and adult T and E2 treatment exposure in adulthood, but future research is needed to determine the molecular mechanisms mediating the observed effects.

## 4. Materials and Methods

### 4.1. Chemicals

Diethylstilbestrol (DES) and bisphenol A (BPA; 99% pure) were obtained from Sigma-Aldrich, St. Louis, MO, USA. Triton X-100 was also purchased from Sigma. Tocopherol-stripped corn oil was from MP Biomedicals (Santa Ana, CA, USA). Silastic tubing (o.d, 0.125 in x i.d. 0.062 in, #508-008) was purchased from Fisher Scientific (San Diego, CA, USA).

### 4.2. Animals

CD-1 mice were originally purchased from Charles River Laboratories (Wilmington, MA, USA), and were maintained as an outbred colony. Mice were housed in standard polypropylene (BPA-free) mouse cages [44] on corncob bedding, with food and water available ad libitum. Prior to pregnancy and after weaning, all animals were fed Purina 5001 maintenance rodent diet; pregnant and lactating females were fed Purina 5008 rodent breeder diet. Drinking water was purified by reverse osmosis and carbon filtration and provided in glass water bottles. Rooms were kept at 23 °C and maintained on a 12 h:12 h light:dark cycle with lights on at 1000 h. Adult females were time-mated by being placed with a stud male for 4 h between 09:00 and 13:00 h, and mating was confirmed by the presence of a copulatory plug; the day of mating was designated as gestation day (GD) 0. Pregnancy was confirmed by weighing females at the time of mating and then again on gestation day 10, just prior to initiation of treatment. The animal study was conducted in full compliance with the rules governing the humane treatment of animals based on the NIH Guide for the Care and Use of Laboratory Animals and protocol 2953 was approved 12-25-08 by the University of Missouri Animal Care and Use Committee (ACUC).

### 4.3. Treatment of Pregnant Females and Neonates

The doses were chosen based on prior published work in our laboratory beginning 25 years ago [4,37]. DES and BPA were dissolved in tocopherol-stripped corn oil. Pregnant mice (*n* = 22/chemical) were dosed with DES at 0.2 ug/kg or BPA at 20 ug/kg in approximately 30 µL volumes once daily from GD 11 to 17 (GD 0 = mating). An electronic micropipetter enabled the delivery of an accurate volume of corn oil and dose of chemical into the mouth [45]; the volume administered was adjusted to maintain a constant dose/kg body weight. Mice readily consume corn oil, and this procedure was used instead of gavage to reduce stress [46]. A vehicle control group of pregnant females (*n* = 6) was treated daily with the same volume of oil without any chemical, and a further unhandled control group (*n* = 8) consisted of animals that remained unhandled throughout pregnancy to control for any possible effects of being handled and fed oil via a micropipetter, since the gavage administration of vehicle led to significant differences from unhandled controls in a rat study [47]. However, we have never found differences in vehicle versus naïve controls in any prior study using our micropipetter administration method. Pregnant females were housed singly on GD 17 and were allowed to deliver and nurse their own offspring. Our decision to stop dosing on GD 17, with delivery on the morning of GD 19 was based on our pharmacokinetic analysis of BPA in CD-1 female mice [45], and our finding that the average level of free (unconjugated) BPA (area under the concentration-time curve or AUC) in the serum of CD-1 mouse fetuses over the 24 h between GD 17 and GD 18 was 8 pg/mL following administration of 20 µg/kg BPA to a pregnant female on GD 17 [41], using the administration procedure described for the present study (maternal AUC serum levels were 12.5-fold higher: 100 pg/mL). BPA doses within this range have led to significant effects on male reproductive system and urethra in male rats and mice, as well as effects on metabolic syndrome [5,22,41,48].

### 4.4. Treatment of Neonatal Males with DES or BPA

On the afternoon of the day of birth on GD 19 (PND 1), litters were weighed, and litters were culled to no more than 14 pups. From PND 1–15, each pup was dosed once daily with DES or BPA dissolved in corn oil as above, at the same doses used for maternal treatment of the litter: DES at 0.2 ug/kg/day and BPA at 20 ug/kg/day. The dosing volume was approximately 1 uL on postnatal day 1, and, as above, the volume was adjusted daily to achieve the correct dose per kg body weight. Dosing was by oral administration; the pup was picked up gently and the tip of a micropipetter was placed into the pup’s mouth. The pups readily drink the solution, and the procedure is less stressful than gavage that involves direct administration into the stomach [46,47,49]. Different groups of control pups (either unhandled or treated with the oil vehicle alone), as well as BPA and DES treated pups, were treated from PND 1 to 15, which was consistent with their mother’s treatment during pregnancy.

### 4.5. Adult Hormone Treatment

Animals were weaned at 22 days old and housed at 1–4 males from the same litter per cage. At 10 weeks old, male mice were implanted subcutaneously with capsules made from Silastic Medical Grade Tubing 0.062” ID, 0.125” OD (Dow Corning 602-285; Fisher Scientific). Each animal received one capsule (10 mm between the capped ends) packed with testosterone and one capsule (5 mm between the capped ends) packed with 17β-estradiol. This procedure provides blood levels of testosterone sufficient to maintain accessory reproductive organ weight within a normal range and was sufficient to stimulate reproductive organ weights in gonadectomized animals from our mouse colony (see Silastic capsule bioassay protocol). Capsules were replaced after 2 months to ensure a sustained release level throughout the 4 months of treatment.

### 4.6. Silastic Capsule Bioassay

In a preliminary study, a small group of gonadectomized adult male and female mice were implanted with either a 10-mm capsule packed with testosterone (males) or a 5-mm capsule packed with estradiol (females); control mice were either not gonadectomized or were gonadectomized and implanted with empty capsules. After two weeks’ treatment, the animals were sacrificed, and the seminal vesicles in males and uterine horns in females were weighed.

### 4.7. Necropsy

All procedures were conducted by an investigator (JAT, MBJ, CLBW and FVS) blind to treatment, with animal ID numbers only decoded for inclusion in different treatment groups after all measurements were made. After 4 months of adult treatment, animals were weighed and euthanized by CO_2_ asphyxiation and cervical dislocation. A subset of animals had to be euthanized and their tissues collected before the formal end of the study due to declining health (bloating and evidence of droplet voiding leading to urine around the genitals). Blood was collected for hormone measurement, and the urogenital tract [including urethra, bladder, seminal vesicles, anterior prostate (coagulating glands), and the rest of the prostate] was briefly fixed in situ before being removed and placed in 10% neutral buffered formalin (NBF). The preputial glands, gonadal (epididymal) fat pads, right testis, right kidney, and brain were weighed, and the right testis was frozen in liquid nitrogen and then stored at −80 °C for later analysis. Selected tissues (prostate, gonadal fat, preputial glands) were also fixed in NBF for later histological examination. Any unusual necropsy findings were recorded in notes and photographed. Fixed tissues were left in 10% NBF overnight and then transferred to 70% ethanol for long-term storage at 4 °C.

### 4.8. External Examination of Fixed Urogenital Tract Specimens

Fixed urogenital tract specimens were examined under a binocular microscope and size was graded by appearance. All grading was performed by a single examiner (FVS) who was blind to sample identities. The size of the prostate, anterior prostate, seminal vesicle and bladder were scored on a four-point scale: (1) Small/normal, (2) Somewhat enlarged, (3) Large, (4) Extremely large. The length and width of the bladder were also recorded using calipers. Bladder volume was estimated using the formula described for calculating egg volume as previously described: Volume = (0.6057 − 0.0018W) * LW^2^, where W = width, L = length [50].

### 4.9. Micro-CT of Urogenital Tract

Urogenital tract specimens were prepared for scanning by immersion in Lugol’s solution overnight for contrast enhancement [51]. Micro-CT scans were performed at the Harry S Truman VA Hospital and University of Missouri Biomolecular Imaging Center, using a Siemens Inveon Micro-SPECT/CT unit (Siemens Pre-Clinical Solutions, Knoxville, TN, USA) with a slice thickness of 84 microns. Raw data files or DICOM files were imported (JAT and AFB) into AMIDE [52]. Measurements of urethra wall 3 mm below the prostate, and bladder wall width at the widest point of the bladder, were made using the software ruler tools. Where possible, one measurement was made in each plane (transverse, coronal, and sagittal) for a total of three measurements, which were averaged to a single measurement. We measured the volume of intact (non-ruptured) bladders from CT scans using the Siemens Inveon software, which permits 3D volume calculations.

### 4.10. Serum Estradiol Assay

Concentrations of estradiol were determined by radioimmunoassay (JAT) using procedures previously validated and described in detail [53]. Briefly, 125-I-labeled estradiol and antiserum were obtained from MP Biomedical (Santa Ana, CA). Unlabeled estradiol was obtained from Steraloids (Wilton, NH, USA). Sensitivity of the assays was 0.11 pg. The intra- and inter-assay coefficients of variation were 5.4% and 12.2%, respectively.

### 4.11. Histology

Prostate and post-prostatic urethra were processed for paraffin infiltration, embedded in paraffin, and sectioned. Sections were deparaffinized, stained with hematoxylin and eosin using standard protocols and examined (blinded to treatment) microscopically by CLBW. Tissue changes were evaluated for severity using a semi-quantitative scoring system.

### 4.12. Daily Sperm Production

Daily sperm production (DSP) was determined by MBJ from the frozen right testis using a procedure previously described [24,48]. Each testis was homogenized for 3 min in 25 mL of physiological saline containing 0.1% Triton X-100 using a semi-micro Waring container on a Waring blender (Fisher Scientific). Aliquots of the homogenate were mixed with Trypan blue, and steps 14–16 (stage II–VIII) spermatids were counted using a hemocytometer. Developing spermatids spend 4.84 days in steps 14–16 during spermatogenesis in the mouse, and thus the number of spermatids per testis and the spermatids per gram of testis were divided by 4.84 to obtain daily sperm production.

### 4.13. Statistical Analysis

Incidence for occurrence of co-pathologies was conducted using the Z-score test for population proportions [54]. Silastic capsule bioassay data were compared with controls by *t*-test. All other data were analyzed by ANOVA, GLM procedure, using SAS version 9.0. The main effect variables were perinatal treatment and adult hormone treatment. However, we first compared perinatal unhandled and oil-vehicle controls, which were then combined into one control (CTL) group. Planned comparisons were made using the LSMeans test when the ANOVA showed statistical significance. Statistical significance was set at *P* < 0.05.

## 5. Conclusions

The results of this study indicate that increased serum estradiol concentration, in conjunction with treatment with enough testosterone to maintain the accessory reproductive organs (thus resulting in an overall increase in the estradiol/testosterone ratio), in adult male mice is associated with increased bladder size due to impaired voiding and, consistently, increased kidney abnormalities, primarily in animals sensitized to these hormones by perinatal exposure to BPA or DES. In addition, although perinatal BPA and DES exposure resulted in increased prostate size independent of adult estrogen treatment, hyperplasia and prostatitis in the dorsal prostate was only seen in histological sections from BPA- and DES-treated animals exposed to adult T+E2 treatment, similar to prior findings regarding the role of developmental and adult estrogen exposure in prostate disease [9,55,56,57]. Thus, these data are consistent with the hypothesis that developmental exposure to low concentrations of xenoestrogens increases adult sensitivity to an increase in the free estrogen to free testosterone ratio during aging in males, which is associated with an increase in the incidence of urethral obstruction and prostate disease.

## Supplementary Materials

Supplementary materials can be found at https://www.mdpi.com/1422-0067/21/11/3902/s1.

## Abbreviations

AR	Androgen receptor
BPA	Bisphenol A
BPH	Benign prostatic hyperplasia
CDC	Centers for Disease Control and Prevention
CT	Computed tomography
DES	Diethylstilbestrol
DOAJ	Directory of open access journals
DSP	Daily sperm production
E2	Estradiol
EE2	Ethinylestradiol
ER	Estrogen receptor
FDA	Food and Drug Administration
GD	Gestation day
MDPI	Multidisciplinary Digital Publishing Institute
NBF	Neutral buffered formalin
OVD	Obstructive voiding disorder
PND	Postnatal day
SHBG	Sex hormone binding globulin
T	Testosterone
UGS	Urogenital sinus
