# Supplementary material for: Interactive Effects of Perinatal BPA or DES and Adult Testosterone and Estradiol Exposure on Adult Urethral Obstruction and Bladder, Kidney, and Prostate Pathology in Male Mice
Source: Int J Mol Sci. 2020 May 30;21(11):3902. doi: 10.3390/ijms21113902 (PMC7313472; doi:10.3390/ijms21113902)
Supplement: Supplementary file 1 [file ijms-21-03902-s001.pdf]

Interactive effects of perinatal BPA or DES and adult testosterone and estradiol exposure on adult urethral obstruction and bladder, kidney, and prostate pathology in male mice

Julia A. Taylor[1], Maren Bell Jones[1], Cynthia L. Besch-Williford[2], Ashley F. Berendzen[3], William A. Ricke[4] and Frederick S. vom Saal[1]

[1] Division of Biological Sciences, University of Missouri, Columbia, MO 65211

[2] IDEXX BioAnalytics, Columbia, MO 65201

[3]Biomolecular Imaging Center, Harry S Truman VA Hospital and University of Missouri, Columbia, MO 65211

[4] George M. O'Brien Center of Research Excellence, Department of Urology, School of Medicine and Public Health, University of Wisconsin, Madison, WI, 53705

Supplemental Materials

Supplemental Figure 1. Body weight and organ weight at necropsy

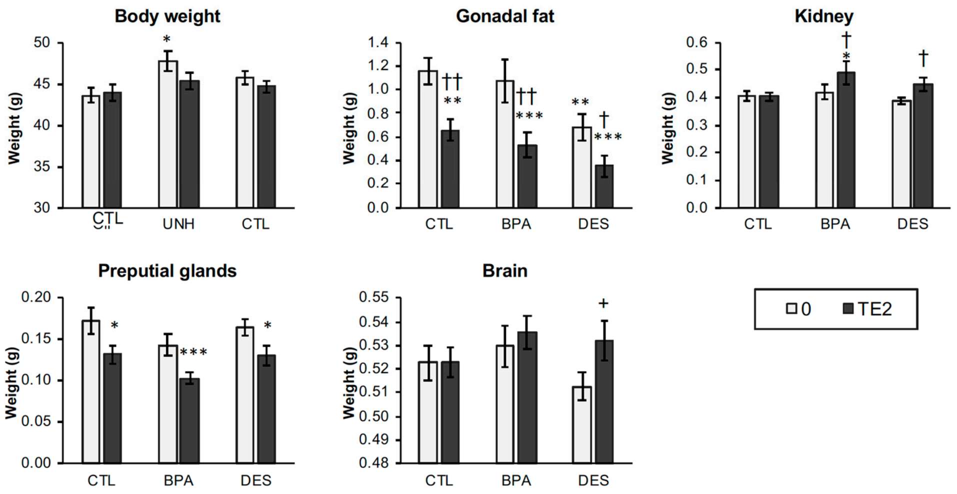

Supplemental Figure 1. Body weights and weights of combined gonadal fat pads, right

kidney, combined preputial glands and the brain at necropsy in CTL-, BPA- and DES-treated mice, with (TE2) and without (0) adult hormone treatment. Values are mean±SEM. *P<0.05, **P<0.01, ***P<0.001 vs. CTL-0;.†P<0.05, ††P<0.01 vs. the same perinatal treatment without T+E2 in adulthood; +P<0.06, DES-TE2 vs. DES-0.

Supplemental Figure 2. Correlation between Micro CT and manual measurement of bladder volume

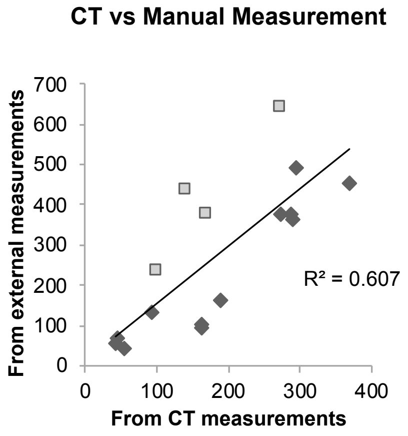

Supplemental Figure 2. Correlation between the different approaches used to measure bladder volume (given in mm^3). CT measurements were made using the imaging software, and calculations based on external dimensions were computed mathematically using the formula [Volume = (0.6057 − 0.0018W) * LW^2], where W = width, L = length. Outliers (samples for which the coefficient of variation between the two measures was over 50%), including some bladders that were wrinkled after fixation, are indicated by

boxed symbols but are included in the calculation of the correlation coefficient. Exclusion of the outlier data points from the calculation results in $R^2 = 0.873$.

Supplemental Figure 3. Preputial glands

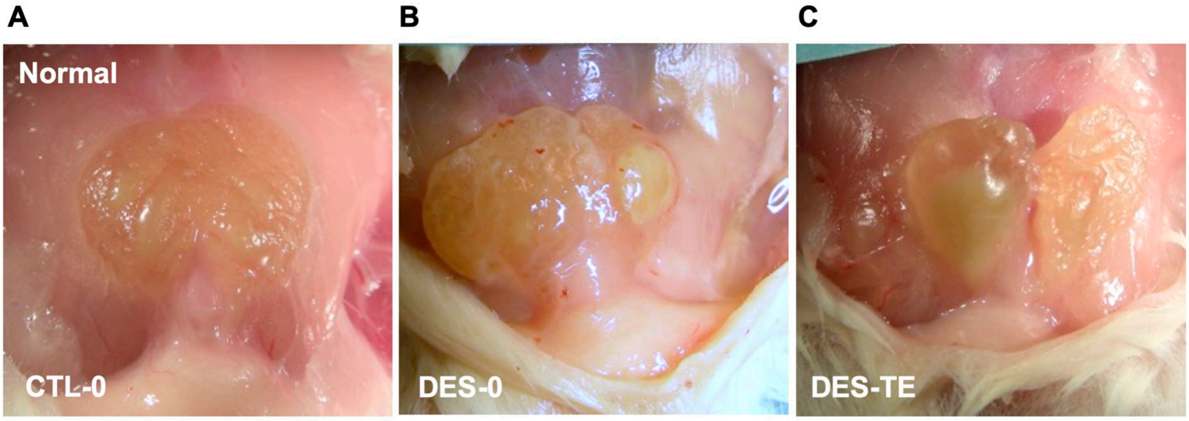

Supplemental Figure 3. Example of preputial gland anomalies, shown for control (CTL-0) and DES treatment groups. A) an example of normal preputial glands in a CTL-0 male, B) a DES-0 male with a small left preputial gland, and C) a DES-TE2 male with a discolored right preputial gland and small left preputial gland. Preputial gland abnormalities were generally worse in T+E2-treated males.

Supplemental Figure 4. Daily sperm production and testis weight

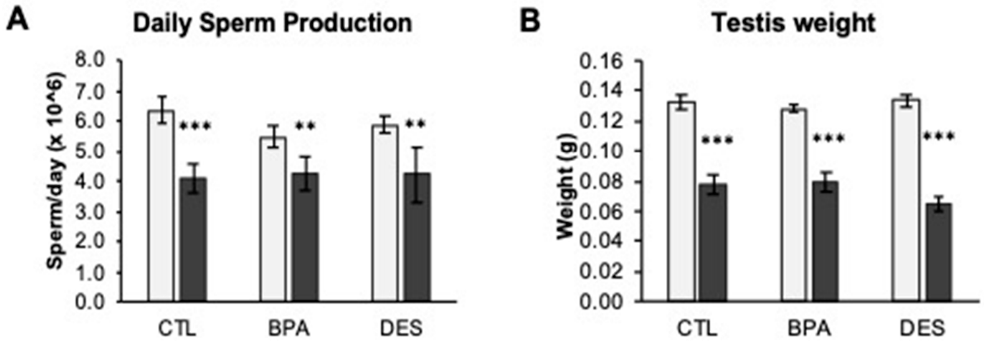

Supplemental Figure 4. A) Daily sperm production and B) weight of right testis in CTL-, BPA- and DES-treated mice, without (0, light bars) and with (TE2, dark bars) adult hormone treatment. Values are mean±SEM.**P<0.01, ***P<0.001 vs. CTL-0.
